# Information Theory Quantifiers in Cryptocurrency Time Series Analysis

**DOI:** 10.3390/e27040450

**Published:** 2025-04-21

**Authors:** Micaela Suriano, Leonidas Facundo Caram, Cesar Caiafa, Hernán Daniel Merlino, Osvaldo Anibal Rosso

**Affiliations:** 1 Departamento de Hidráulica, Facultad de Ingeniería, Universidad de Buenos Aires, Av. Las Heras 2214, Buenos Aires C1127AAR, Argentina; 2 Laboratorio de Redes y Sistemas Móviles, Departamento de Electrónica, Facultad de Ingeniería, Universidad de Buenos Aires, Buenos Aires C1063ACV, Argentina; fcaram@fi.uba.ar; 3 Instituto Argentino de Radioastronomía-CCT La Plata, CONICET/CIC-PBA/UNLP, Camino Gral. Belgrano Km 40, Berazategui B1894XAB, Argentina; ccaiafa@gmail.com; 4 Grupo IngenIA, Facultad de Ingeniería, Universidad de Buenos Aires, Buenos Aires C1063ACV, Argentina; hmerlino@fi.uba.ar; 5 Instituto de Física (IFLP), Universidad Nacional de La Plata, CONICET, La Plata B1900AJJ, Argentina; oarosso@gmail.com; 6 Instituto de Física, Universidade Federal de Alagoas (UFAL), Maceió 57072-970, Brazil

**Keywords:** permutation entropy, statistical complexity, cryptocurrency

## Abstract

This paper investigates the temporal evolution of cryptocurrency time series using information measures such as complexity, entropy, and Fisher information. The main objective is to differentiate between various levels of randomness and chaos. The methodology was applied to 176 daily closing price time series of different cryptocurrencies, from October 2015 to October 2024, with more than 30 days of data and not completely null. Complexity–entropy causality plane (CECP) analysis reveals that daily cryptocurrency series with lengths of two years or less exhibit chaotic behavior, while those longer than two years display stochastic behavior. Most longer series resemble colored noise, with the parameter *k* varying between 0 and 2. Additionally, Natural Language Processing (NLP) analysis identified the most relevant terms in each white paper, facilitating a clustering method that resulted in four distinct clusters. However, no significant characteristics were found across these clusters in terms of the dynamics of the time series. This finding challenges the assumption that project narratives dictate market behavior. For this reason, investment recommendations should prioritize real-time informational metrics over whitepaper content.

## 1. Introduction

The relationship between complex systems and cryptocurrencies has been a topic that has captured the interest of the scientific community specializing in cryptocurrencies in recent years. This interest was further amplified by the COVID-19 pandemic, as [1] found an increase in volatility during this period. This is hypothetically due to the time that more people were dedicated to trading cryptocurrencies, especially Bitcoin, as more individuals engaged in trading during lockdowns.

This issue was addressed through the analysis of entropy by [2], concluding that the pandemic altered the volatility inherent in cryptocurrency markets. A study by [3] analyzed the increase in entropy in periods of turbulence, such as during the COVID-19 pandemic when the entire market showed increased volatility, rather than isolated cryptocurrencies. Paradoxically [4] observed volatility consolidation in mature cryptocurrencies, suggesting market maturation phases where entropy metrics require multi-scale approaches.

In [5], the authors employed the Fisher information measure alongside permutation entropy to assess the evolving informational efficiency and price disorder dynamics of five major cryptocurrencies across pre- and intra-pandemic periods, offering critical insights into their maturity and predictability for liquidity risk diversification strategies.

Studies have identified persistent chaos patterns in these markets, proposing alternative methods for volatility analysis [6]. Incorporating sentiment analysis improves volatility analysis in chaotic markets [7]. The unpredictability of cryptocurrency markets reflects a chaotic system with high entropy [8].

The authors in [9] examined how entropy, as a variable in the analysis of network decentralization, contributes to market volatility. The intrinsic volatility of cryptocurrency markets renders traditional volatility evaluation methods inadequate [10]. Research on market risk using high-frequency entropy concluded that this method is a good predictor of Bitcoin’s value risk [11]. Entropy is also applied as a measure of uncertainty in portfolio management [12], showing that portfolio diversification is a reasonable practice for reducing return uncertainty. The complexity–entropy causality plane (CEPC) analysis method has been used to distinguish stages of stock market development [13] and to analyze algorithmic behavior in cryptocurrencies [14], serving as a precedent for complexity-based approaches in this field.

Previous studies have highlighted the utility of clustering in cryptocurrency analysis, such as the work of [15], which identified temporal efficiency patterns using permutation entropy and statistical complexity. Our approach extends this paradigm by applying clustering techniques to whitepaper analysis, offering a complementary perspective focused on project fundamentals rather than market metrics.

This paper analyzes the temporal evolution of different cryptocurrency time series, utilizing information quantifiers to map the complexity–entropy causality plane (CECP) and describe system dynamics. By combining global statistical complexity and local Fisher information measures, we classify clusters based on whitepaper attributes, which group projects according to their declared rules and governance structures. These clusters are then evaluated using information theoretic metrics to identify links between project frameworks and statistical market behavior.

This paper is structured as follows:

Materials and Methods: description of applied methods, equations, data sources, and time series characteristics;

Results and Discussion: interpretation of outcomes and analysis;

Conclusions: summary of key findings.

## 2. Materials and Methods

### 2.1. Ordinal Patterns

Cryptocurrencies, like many other economic phenomena, produce time series observations. To describe the nature of the processes, it is necessary to determine the appropriate probability density function associated with the analyzed time series. For this analysis, the probability distribution function ***P*** can be determined using the method of ordinal patterns developed by Bandt and Pompe (2002) [16]. This method replaces the values that appear in the time series with their corresponding range sequence. This procedure considers the temporal causality within the dynamics of the process.

For a given time series {*x_t_*: *t* = 1, …, *N*}, the delay time τ (τ ϵ ℕ), an embedding dimension *D* ≥ 2 (*D* ϵ ℕ), and the *D*-ordinal pattern are defined by

(1) $$s\to \left\{X_{s-\left(D-1\right)\tau },X_{s-\left(D-2\right)\tau },\ldots ,X_{s-\tau },X_{s}\right\}$$

A *D*-dimensional vector is assigned for every time instant *s*, and it is determined from the evaluation of the time series in the *s − (D −* 1*) τ*, *…*, *s − τ*, *s* instants. The *D*-ordinal patterns related to the instant *s* are referred to as the permutation ***π*** = {*r*_0_*, r*_1_*, …, r_D−_*_1_} of {0, 1, …, *D −* 1} described by

(2) $$x_{s-r_{0}\tau }\geq x_{s-r_{1}\tau }\geq \ldots \geq x_{s-r_{D}-2\tau }\geq x_{s-r_{D}-1\tau }.$$

To find a unique result, $r_{i}<r_{i-1}\;\mathrm{if}\;x_{s-r_{i}\tau }<x_{s-r_{i-1}\tau }$ is considered and equal consecutive values are not usual. The vector defined by Equation (2) becomes the unique symbol **π**.

**Figure 1:**
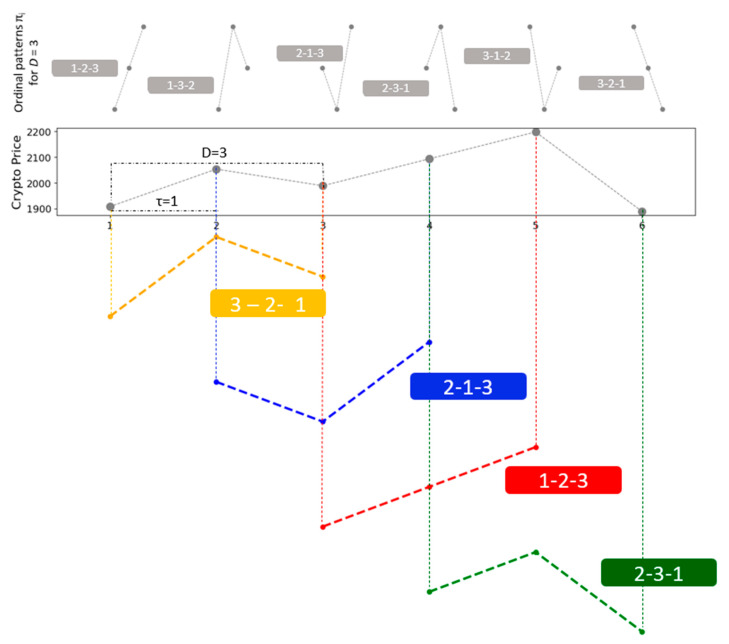
An illustration of identifying ordinal patterns to a cryptocurrency time series (dimension *D* = 3 and an embedding delay *τ* = 1). At the top, the resultant ordinal patterns *π*_i_ for *D* = 3 are presented. At the bottom, the methodology applied to a cryptocurrency time series with *τ* = 1 is found. The pattern is obtained by replacing the original values with their corresponding rankings, (adapted from [17,18]).

Figure 1 illustrates the determination of the permutations **π_i_** for *D* = 3 with a time lag *τ* = 1. At the top of the figure the possible ordinal patterns **π_i_** for *D* = 3 are presented, while the analysis of a cryptocurrency time series with *τ* = 1 is displayed at the bottom. For each possible permutation **π_i_** derived from the order *D*!, the associated relative frequencies can be calculated by counting how many times each sequence appears in the series and dividing that number by the total number of sequences. This process yields the probability distribution of the ordinal patterns for the given time series.

The selection of the value of *D* should correspond to the minimum sampling frequency needed to capture all the information about the signal’s time structure [19]. To differentiate between deterministic and stochastic dynamics, it is recommended that *N* ≫ *D*!, where *N* denotes the length of the time series [20]. Bandt and Pompe [16] proposed 3 ≤ *D* ≤ 7 and *τ* = 1. The embedding delay *τ* = 1 captured the daily temporal structure of the cryptocurrency time series.

### 2.2. Complexity–Entropy Causality Plane (CECP)

The Shannon logarithmic information measure *S[**P**]* is considered a measure of the uncertainty associated with a phenomenon described by ***P,*** with ***P*** being a given arbitrary probability distribution ***P*** = {*p_i_*: *i* = 1, …, *N*}, and determined by

(3) $$S\left[P\right]=-\sum_{i=1}^{N}p_{i}\ln\left(p_{i}\right)$$

On the one hand, when $S\left[P\right]$ = 0, it is possible to predict with certainty which of the possible scenarios *i*, associated with probabilities *p_i_*, will occur. On the other hand, when ***P*** is the uniform distribution, our ignorance is maximum. A measure of statistical complexity [21] capable of detecting key details of the dynamics is defined through the product

(4) $$C_{JS}\left[P\right]=Q_{J}\left[P,P_{e}\right]{\cdot H}_{S}\left[P\right]$$

and the generalized Shannon entropy [22] is

(5) $$H_{S}=\frac{S\left[P\right]}{S_{máx}}$$

with *S_máx_* = *S*[***P_e_***] *= ln*(*N*), 0 ≤ *H*_s_ ≤ 1 and ***P_e_*** *=* 1*/N*, *…*, 1*/N* the uniform distribution.

The disequilibrium is defined in terms of the Jensen–Shannon divergence:

(6) $$Q_{j}\left[P,P_{e}\right]=Q_{0}J\left[P,P_{e}\right]$$

with *Q*_0_ being a normalization constant equal to the inverse of the maximum possible value of *J*[***P***,***P_e_***] and the Jensen–Shannon divergence:

(7) $$J\left[P,P_{e}\right]=S\left[\frac{P+P_{e}}{2}\right]-\frac{S\left[P\right]}{2}-\frac{S\left[P_{e}\right]}{2}$$

**Figure 2:**
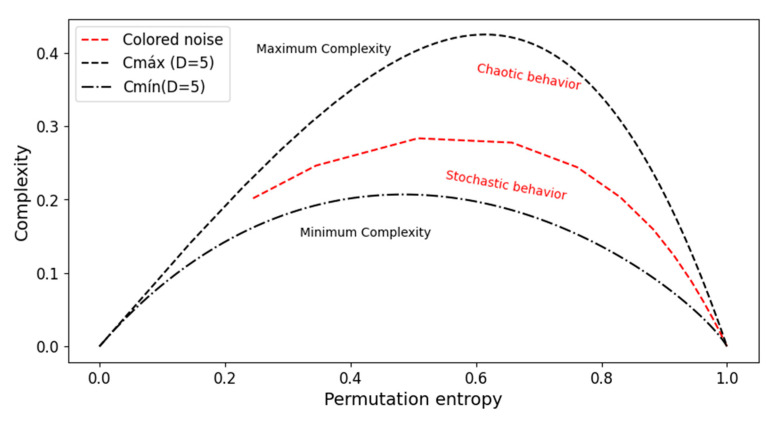
Illustrative representation of the complexity–entropy causality plane (CECP) [13]. In the dashed line are represented the maximum and minimum boundaries for *D* = 5, as well in red the colored noise with the approximate identification of chaotic and stochastic zones [18].

The complexity–entropy causality plane (CECP) represents the plot of the permutation statistical complexity *C_JS_* versus the generalized Shannon entropy *H_S_*, including the bounds that determine the admissible region which depends solely on the embedding dimension *D.* The maximum and minimum envelope complexity can be calculated as a function of the entropy [23]. Figure 2 provides a schematic illustration of the CECP. The maximum and minimum boundaries are calculated for *D* = 5 (denoted *C_máx_* and *C_mín_*, respectively) along with an approximate classification of chaotic and stochastic zones.

### 2.3. Fisher’s Information Measure (FIM)

The Fisher–Shannon plane is another graphic representation used to identify the characteristics of the time series dynamics based on the probability distribution ***P***. This approach can reveal the informational characteristics of the planar position [24]. The Fisher’s information measure (FIM) [25] represents a measure of the ability to estimate the amount of information that can be extracted from a set of measurements [26] and *F* serves as a measure of the gradient content of the distribution *f*(*x*), calculated by

(8) $$F\left[f\right]=\int_{∆}\frac{1}{f\left(x\right)}{\left[\frac{df\left(x\right)}{dx}\right]}^{2}dx=4\int_{∆}{\left[\frac{d\Psi \left(x\right)}{dx}\right]}^{2}$$

For a discrete environment, the best-behaved expression to use [27] is the discrete normalized FIM, as obtained by

(9) $$F\left[P\right]=F_{0}\sum_{i=1}^{N-1}{\left[{\left(p_{i+1}\right)}^{\frac{1}{2}}-{\left(p_{i}\right)}^{\frac{1}{2}}\right]}^{2}$$

and the normalization constant *F*_0_ is obtained by

(10) $$F_{0}=\left\{\begin{array}{c} 1\;if\;p_{i*}=1\;for\;i^{*}=1\;or\;i^{*}=N\;and\;p_{i}=0\;\forall i\neq i^{*}\\ 0\;otherwise\; \end{array}\right\}$$

Then, the Fischer–Shannon causality plane Hs x F can be calculated, where Hs is the generalized Shannon entropy in Equation (3).

### 2.4. Clustering

Cryptocurrencies can be described based on their white papers, which are technical documents that outline the project, its value proposition, the underlying technology, and other relevant details. A white paper is similar to a business plan and informs investors and users about the project.

By applying Natural Language Processing (NLP) analysis, it is possible to identify the most relevant terms in each whitepaper, allowing us to apply a clustering method that results in four clusters. Consequently, each token is assigned four membership coefficients indicating the degree of similarity with each cluster.

To identify clusters, the Latent Dirichlet Allocation (LDA) model was used. LDA is a probabilistic model that attempts to generate clusters based on the similarities between documents [28]. The basic concept behind this clustering is that similar documents are grouped into the same cluster, implying that cryptocurrencies with comparable characteristics should exhibit similar behavior.

### 2.5. Cryptocurrency Time Series

This paper applied the methods described to 176 daily closing price time series of different cryptocurrencies. The data were obtained from CoinMarketCap (coinmarketcap.com) (accessed on 15 October 2024). The data selection was based on the length of the series, spanning from October 2015 to October 2024, requiring series of more than 30 days in length and not completely null. A second classification was conducted to compare the behavior of series with a length of two years or less to those of greater size, as detailed in Table A1 and Table A2, respectively. In [29], the authors suggest exercising caution when applying permutation-based entropies to short high-frequency variability series characterized by a low signal-to-noise ratios. However, daily closing price time series are analyzed in this study, which smooths out intraday fluctuations and, therefore, is not considered to be significantly affected by high-frequency noise.

The algorithms used for analysis were obtained from the Python3.11.12 library ordpy 1.2.0 [30] to apply the methodology to the data. The color noise series was generated with the library colorednoise 2.2.0 (by Felix Patzel), which generates Gaussian distributed noise with a power law spectrum based on the algorithm [31]. Plots are created using the Matplotlib 3.10.0 [32], seaborn 0.13.2 [33], and GeoPandas 1.0.1 [34] Python libraries.

## 3. Results and Discussion

The results were obtained by applying the methodology to the daily closing price time series of different cryptocurrencies, as detailed in Appendix A. The data sets were divided into two groups: those with lengths of two years or less and those with lengths longer than two years. Bandt and Pompe [16] proposed 3 ≤ *D* ≤ 7 and *τ* = 1. In [35] it was found that an embedding dimension 3 ≤ *D* ≤ 5, as well as a window length of about 10^3^ sampling points, is favorable. Moreover, increasing the value of *D* results in a greater incorporation of temporal causality into the embedding vectors [14]. In this case, the embedding delay is considered to be *τ* = 1 in order to capture the daily temporal structure of the cryptocurrency time series. The embedding dimension is set to *D* = 5 to effectively capture the variability observed within a typical working week in cryptocurrency markets. This choice enables the construction of embedding vectors that represent five consecutive daily observations, aligning with the standard trading cycle and enabling the model to incorporate temporal dependencies and weekly behavioral patterns specific to cryptocurrencies. Since the lengths of the series are variable, considering an average length *Na* = 1950, *D* = 5 satisfied the requirement that *Na* ≫ *D*! [20].

The methodology was applied to dynamic stochastic series of *k*-noises (noise with a power spectrum frequency dependence characterized by $f^{\left(-k\right)}$), where *k* ranged from 0.00 to 3.50 in increments of 0.25, with 10 random simulations conducted for each *k* value.

**Figure 3:**
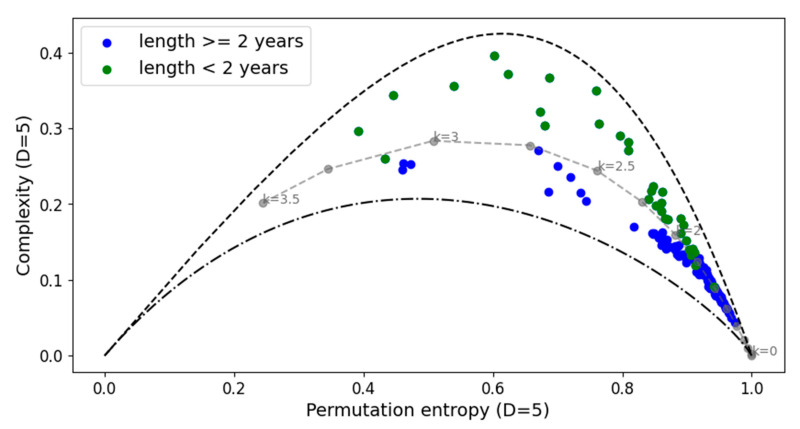
Complexity–entropy causality plane analyses for all the cryptocurrency series and noise with power spectrum frequency-varying parameter *k* (gray dashed line). In color blue the time series with lengths of more than two years and in green those with equal two years or less. The parameters used are *D* = 5 and *τ* = 1.

**Figure 4:**
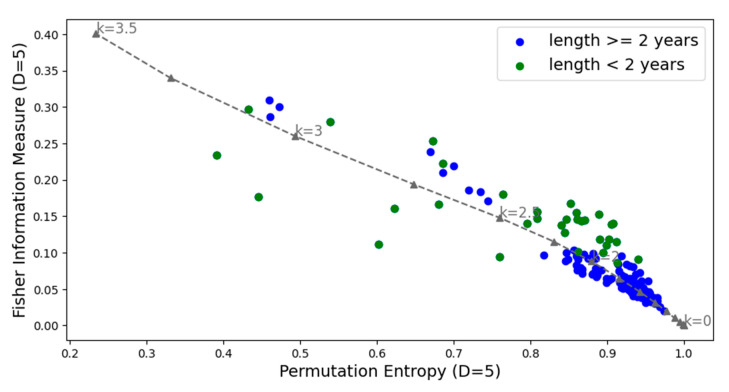
Fisher information measures and permutation entropy planes for all the cryptocurrency series analyzed and noise with power spectrum frequency-varying parameter *k* (gray dashed line). In color blue the time series with a length of more than two years and in green those with equal two years or less. The parameters used are *D* = 5 and *τ* = 1.

The complexity–entropy causality planes (CECPs) using Shannon entropy and the Fischer–Shannon plane for the daily cryptocurrency time series are illustrated in Figure 3 and Figure 4, respectively.

The CECP analyses showed that, in most cases, daily cryptocurrency time series with lengths of two years or less exhibit chaotic behavior, while those longer than two years display stochastic behavior. For longer duration series, it can be observed that colored noise represents an upper bound on the CECP.

**Figure 5:**
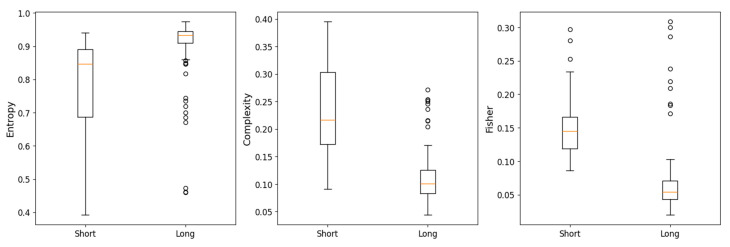
Box plots to compare short (equal or less than two years) and long (more than two years) cryptocurrency time series in terms of entropy, complexity and Fisher parameters. The parameters used are *D* = 5 and τ = 1.

As shown in Figure 5, in all instances, the complexity, entropy, and Fisher parameters exhibit significantly greater variability in the short series compared to the long series. The long series demonstrate more stable parameter values, with a higher median entropy, lower complexity, and lower Fisher values when compared to the short series.

Subsequently, a clustering analysis was conducted for the subgroup of cryptocurrencies identified based on the characteristics outlined in the white papers across four topics. After applying the Latent Dirichlet Allocation (LDA) algorithm, the resulting clusters are as follows:

Topic 1 has 51 cryptocurrency time series: ‘ADC’, ‘ANC’, ‘ARG’, ‘BITS’, ‘BLOCK’, ‘BSD’, ‘BTB’, ‘CANN’, ‘CLAM’,’CSC’, ‘CURE’, ‘DEM’, ‘EMD’, ‘ETH’, ‘FLT’, ‘FRC’, ‘GRC’, ‘IFC’, ‘IXC’, ‘LOG’, ‘NAV’, ‘NET’, ‘NOBL’, ‘NTRN’, ‘NVC’, ‘OMNI’, ‘ORB’, ‘PINK’, ‘POP’, ‘PTC’, ‘RBT’, ‘RDD’, ‘RED’, ‘SLG’, ‘SMLY’, ‘THC’, ‘TRK’, ‘TRUST’, ‘TTC’, ‘USNBT’, ‘UTC’, ‘VIA’, ‘XCN’, ‘XDN’, ‘XLM’, ‘XMR’, ‘XPD’, ‘XPM’, ‘XQN’, ‘XST’, and ‘XVG’.

Topic 2 has 45 cryptocurrency time series: ‘ARI’, ‘BBR’, ‘BLU’, ‘BTC’, ‘BTS’, ‘CLOAK’, ‘CRW’, ‘CRYPT’, ‘DIME’, ‘DMD’, ‘DP’, ‘DTC’, ‘EFL’, ‘EMC2’, ‘FJC’, ‘FTC’, ‘GLD’, ‘GRS’, ‘HBN’, ‘LDOGE’, ‘LOG’, ‘MAX’, ‘MEC’, ‘NOTE’, ‘NYC’, ‘POT’, ‘PPC’, ‘PXC’, ‘RBY’, ‘SKC’, ‘SLR’, ‘SOON’, ‘STV’, ‘SXC’, ‘TGC’, ‘TIPS’, ‘UBQ’, ‘UNIT’, ‘UNO’, ‘VTC’, ‘XBC’, ‘XCO’, ‘XMY’, ‘XPY’, and ‘XWC’.

Topic 3 has 13 cryptocurrency time series: ‘AC’, ‘BSTY’, ‘DGB’, ‘DGC’, ‘GAME’, ‘GRN’, ‘PHO’, ‘PLNC’, ‘TES’, ‘TROLL’, ‘VRC’, ‘WDC’, and ‘XCP’.

Topic 4 has 30 cryptocurrency time series: ‘42’, ‘ACOIN’, ‘AIB’, ‘ANC’, ‘AUR’, ‘BAY’, ‘BCN’, ‘BLC’, ‘BTA’, ‘BTCD’, ‘CASH’, ‘DON’, ‘DOPE’, ‘EMC’, ‘ENRG’, ‘FAIR’, ‘FLO’, ‘GP’, ‘IOC’, ‘KOBO’, ‘MINT’, ‘MONA’, ‘NXS’, ‘NXT’, ‘OK’, ‘PND’, ‘SAK’, ‘SUPER’, ‘SYS’, and ‘VIA’.

**Figure 6:**
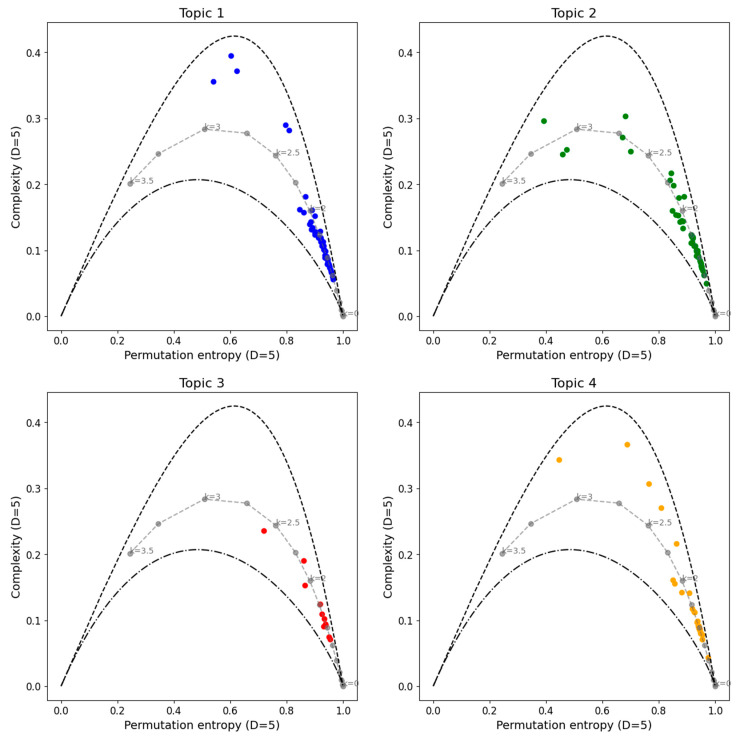
Complexity–entropy causality plane analyses for the cryptocurrency series divided in clusters and noise with power spectrum frequency-varying parameter *k* (gray dashed line). Topic 1 in color blue, Topic 2 in color green, Topic 3 in color red and Topic 4 in color orange. The parameters used are *D* = 5 and *τ* = 1.

Figure 6 presents the CECP analyses for clustering classification. It can be observed that Topics 1 and 2 exhibit similar behaviors. Most series are concentrated near the noise curve between *k* = 0 and *k* = 2, displaying chaotic behavior for lower entropy values. For Topic 2, the majority of the series are close to the noise zone region between *k* = 0 and *k* = 2.5, with some values bordering the curve for lower entropy values. Finally, for Topic 3, the values appear near the noise curve between *k* = 0 and *k* = 2.5, but it should be noted that the sample size is smaller than in the other cases, with only 13 cases analyzed.

**Figure 7:**
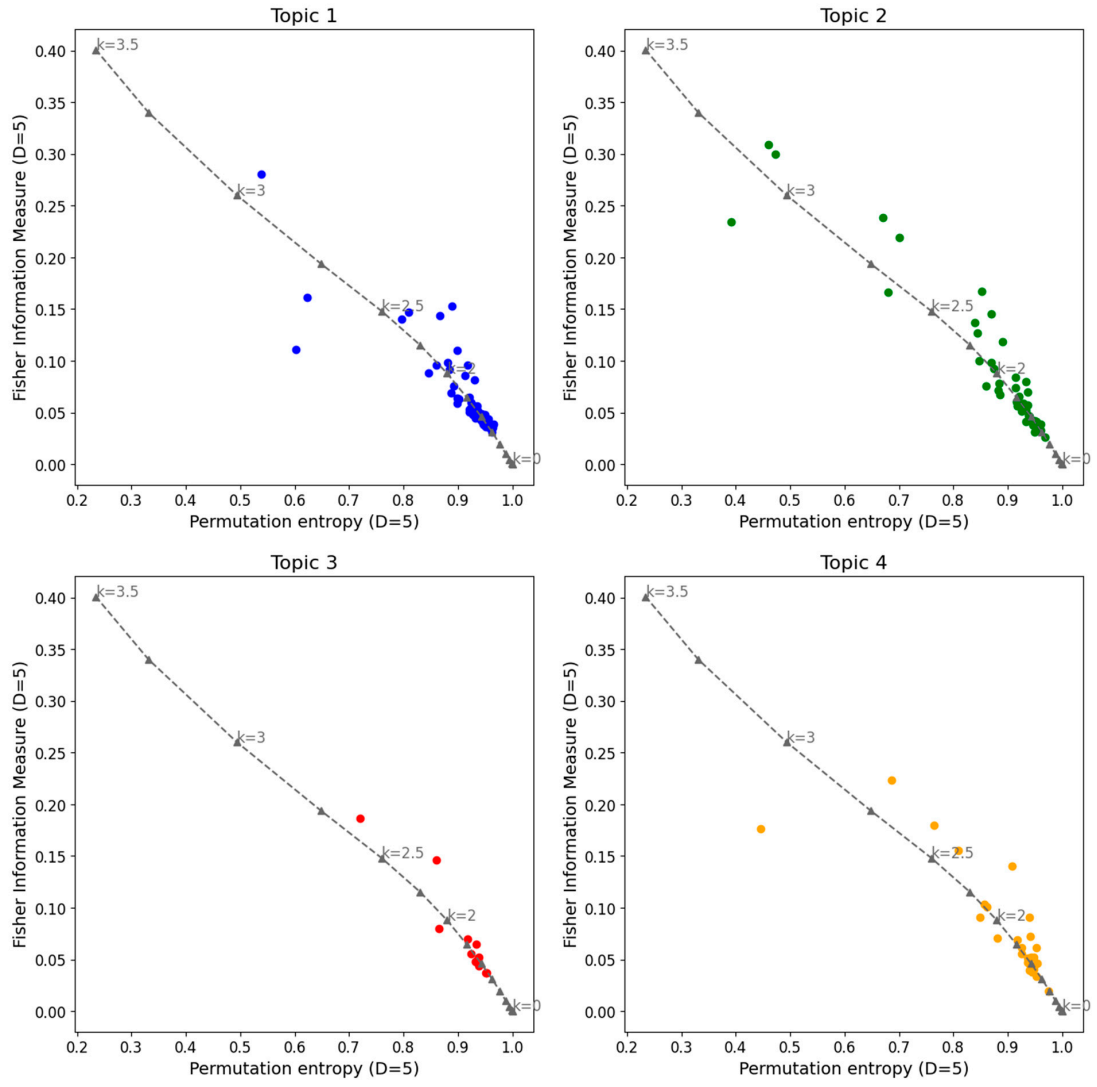
Fisher information measures and permutation entropy planes for all the cryptocurrency series analyzed and noise with power spectrum frequency-varying parameter *k* (gray dashed line). Topic 1 in color blue, Topic 2 in color green, Topic 3 in color red, and Topic 4 in color orange. The parameters used are *D* = 5 and τ = 1.

The Fisher information measure vs. permutation entropy plane is shown in Figure 7, comparing the different topics identified in this study. No significant differences in behavior are observed between topics. In all cases, most series are concentrated near the noise curve between *k* = 0 and *k* = 2.5, with greater dispersion for higher *k* values.

**Figure 8:**
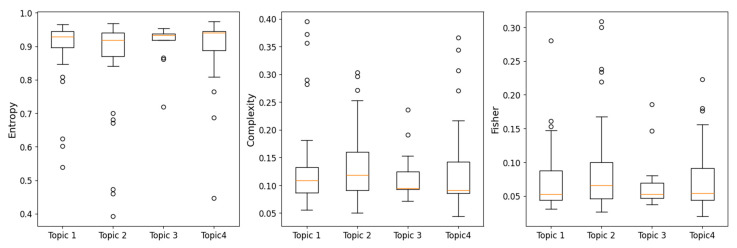
Boxplots to compare the different topics clustering of the cryptocurrency time series in terms of entropy, complexity and Fisher parameters. The parameters used are *D* = 5 and τ = 1.

In Figure 8, the box plots compare the different topics in terms of parameters such as entropy, complexity, and Fisher information. The graphs show that no significant differences exist in the mean values.

The clustering technique successfully identified four distinct groups based on the main characteristics in their white papers. However, these differences were not significant when evaluated using information measures such as entropy, complexity, and Fisher information. Despite the variations in the white papers, the time series dynamics remained similar. This result reveals the limited predictive power of white papers, suggesting that declared project rules do not directly govern observed market efficiency or complexity.

## 4. Conclusions

The temporal evolution of cryptocurrency time series was analyzed using information measures. The dynamics of these series were described in terms of complexity, entropy, and Fisher information. The main objective was to distinguish between different levels of randomness and chaos.

The representation in the complexity-entropy causality plane (CECP) indicates that daily cryptocurrency series with a length of two years or less exhibit chaotic behavior, while series longer than two years are associated with stochastic behavior. Most of the longer series display characteristics similar to colored noise, with the parameter *k* varying between 0 and 2. The CECP framework could help investors distinguish between speculative assets (chaotic phases) and mature markets (stochastic phases), informing portfolio diversification strategies.

By applying Natural Language Processing (NLP) analysis, the most relevant terms in each whitepaper were identified, enabling the use of a clustering method that resulted in four distinct clusters. However, no significant characteristics were found in terms of information measures, challenging the assumption that project narratives dictate market behavior. For this reason, an investment recommendation should prioritize real-time informational metrics over white paper content.

Future research should investigate other variables, including technological innovations, investor sentiment metrics, and macro-financial drivers (regulatory shifts and cross-asset correlations), that may be influencing the behavior of time series in cryptocurrencies.

Understanding these dynamics is critical for constructing diversified portfolios that mitigate risks associated with chaotic market phases while capitalizing on efficiency regimes. For instance, identifying assets transitioning from chaos to stochasticity—via complexity-entropy metrics—can inform strategic asset allocation, balancing high-volatility cryptocurrencies with stable, entropy-resilient ones.

## Data Availability

The data used to support the findings from this study are included within the article. The processed data are available from the corresponding author upon request.

## Appendix A

In this appendix, the information from the cryptocurrency time series is shown. Table A1 shows the series with lengths of two years or less and Table A2 shows those of greater length.

**Table A1:** Information from the cryptocurrency time series with two years or less length.

Crypto	Length	Start	End
ARI	927	20/12/2021	3/7/2024
BAY	250	1/11/2021	8/7/2022
BCF	298	25/5/2022	18/3/2023
BTCD	227	19/11/2023	2/7/2024
BTCS	301	19/12/2023	14/10/2024
CBX	1075	5/11/2021	14/10/2024
CRYPT	563	10/10/2021	25/4/2023
DON	992	28/8/2020	28/5/2023
DP	342	22/11/2021	31/10/2022
FCN	88	7/10/2021	3/1/2022
FLO	317	10/3/2021	20/1/2022
FLT	204	25/3/2024	14/10/2024
GLD	573	14/12/2021	9/7/2023
GP	45	1/12/2021	14/1/2022
HYPER	487	27/4/2021	11/9/2022
NET	58	8/3/2023	4/5/2023
NOBL	182	16/4/2024	14/10/2024
PND	136	28/3/2022	10/8/2022
PTC	95	12/7/2024	14/10/2024
RPC	450	24/1/2022	30/8/2023
SAK	373	8/9/2022	15/9/2023
SLG	720	19/7/2022	7/7/2024
SLR	434	18/11/2021	29/1/2023
SOON	505	14/10/2022	1/3/2024
SRC	71	5/8/2024	14/10/2024
TES	430	9/8/2023	14/10/2024
TGC	99	4/1/2022	12/4/2022
TIT	353	23/6/2023	9/6/2024
TRI	376	15/12/2020	27/12/2021
TRK	57	30/11/2021	2/2/2022
TRUST	755	31/7/2020	24/8/2022
TTC	712	29/7/2022	9/7/2024
UNB	1036	14/12/2021	14/10/2024
UTC	408	22/3/2022	5/6/2023

**Table A2:** Information from the cryptocurrency time series with lengths of more than two years.

Crypto	Length	Start	End
42	2532	9/11/2017	14/10/2024
AC	1410	13/10/2020	25/8/2024
ACOIN	2527	9/11/2017	14/10/2024
ADC	2532	9/11/2017	14/10/2024
AIB	1998	9/11/2017	13/6/2023
ANC	2532	9/11/2017	14/10/2024
ARG	1162	10/8/2021	14/10/2024
AUR	2532	9/11/2017	14/10/2024
BBR	1656	27/11/2017	9/6/2022
BCN	2531	9/11/2017	14/10/2024
BITB	2532	9/11/2017	14/10/2024
BITS	2518	9/11/2017	14/10/2024
BLC	1537	9/11/2017	23/1/2022
BLK	2532	9/11/2017	14/10/2024
BLOCK	2532	9/11/2017	14/10/2024
BLU	2532	9/11/2017	14/10/2024
BSD	2284	9/11/2017	9/2/2024
BSTY	2532	9/11/2017	14/10/2024
BTA	2532	9/11/2017	14/10/2024
BTB	2105	10/1/2019	14/10/2024
BTC	3285	18/10/2015	14/10/2024
BTS	2532	9/11/2017	14/10/2024
C2	2526	9/11/2017	14/10/2024
CANN	2532	9/11/2017	14/10/2024
CASH	1637	22/4/2020	14/10/2024
CCN	2055	1/3/2019	14/10/2024
CLAM	2532	9/11/2017	14/10/2024
CLOAK	2532	9/11/2017	14/10/2024
CRW	2532	9/11/2017	14/10/2024
CSC	2500	9/11/2017	14/10/2024
CURE	2532	9/11/2017	14/10/2024
DASH	2532	9/11/2017	14/10/2024
DEM	2461	9/11/2017	14/10/2024
DGB	2532	9/11/2017	14/10/2024
DGC	3285	18/10/2015	14/10/2024
DIME	2532	9/11/2017	14/10/2024
DMD	2532	9/11/2017	14/10/2024
DOGE	2532	9/11/2017	14/10/2024
DOPE	2532	9/11/2017	14/10/2024
DTC	2237	21/2/2018	14/4/2024
ECC	1762	9/11/2017	5/9/2022
EFL	2120	9/11/2017	14/10/2024
EMC	2532	9/11/2017	14/10/2024
EMC2	2222	9/11/2017	12/12/2023
EMD	1573	9/11/2017	10/3/2022
ENRG	1601	9/11/2017	30/6/2022
ETH	2532	9/11/2017	14/10/2024
EXCL	1871	9/11/2017	23/12/2022
FAIR	1154	9/11/2017	10/5/2022
FJC	1689	9/11/2017	8/7/2022
FRC	3181	18/10/2015	14/10/2024
FST	1775	8/4/2019	15/2/2024
FTC	3285	18/10/2015	14/10/2024
GAME	2532	9/11/2017	14/10/2024
GCN	2104	9/11/2017	16/1/2024
GRC	2284	9/11/2017	9/2/2024
GRN	1922	21/5/2018	24/8/2023
GRS	2532	9/11/2017	14/10/2024
HBN	1821	9/11/2017	14/10/2024
IFC	2012	9/11/2017	14/10/2024
IOC	1673	9/11/2017	8/6/2022
IXC	3208	18/10/2015	14/10/2024
KOBO	2524	9/11/2017	14/10/2024
LDOGE	996	17/4/2020	14/10/2024
LOG	1795	9/11/2017	14/10/2024
LTC	3285	18/10/2015	14/10/2024
MAX	2531	9/11/2017	14/10/2024
MEC	2533	18/10/2015	2/11/2023
MINT	2284	9/11/2017	9/2/2024
MNC	1301	21/2/2019	13/9/2022
MONA	2532	9/11/2017	14/10/2024
MUE	1674	9/11/2017	9/6/2022
NAV	2532	9/11/2017	14/10/2024
NLG	1839	9/11/2017	21/11/2022
NMC	3285	18/10/2015	14/10/2024
NOTE	2312	9/11/2017	3/5/2024
NTRN	2532	9/11/2017	14/10/2024
NVC	3002	18/10/2015	14/10/2024
NXS	2333	9/11/2017	14/10/2024
NXT	2532	9/11/2017	14/10/2024
NYAN	1206	1/9/2018	14/2/2023
NYC	2522	9/11/2017	14/10/2024
OK	2532	9/11/2017	14/10/2024
OMNI	2532	9/11/2017	14/10/2024
ORB	2051	9/11/2017	24/6/2023
PHO	1516	9/11/2017	23/1/2022
PIGGY	1097	14/10/2021	14/10/2024
PINK	2284	9/11/2017	9/2/2024
PLNC	2496	9/11/2017	14/10/2024
POP	1466	10/10/2020	14/10/2024
POT	2529	9/11/2017	14/10/2024
PPC	3285	18/10/2015	14/10/2024
PXC	3071	18/10/2015	14/10/2024
PXI	2532	9/11/2017	14/10/2024
QRK	2530	9/11/2017	14/10/2024
RBT	2532	9/11/2017	14/10/2024
RBY	2532	9/11/2017	14/10/2024
RDD	2532	9/11/2017	14/10/2024
RED	2337	23/5/2018	14/10/2024
SKC	1356	4/5/2020	19/1/2024
SMC	1667	9/11/2017	2/6/2022
SMLY	2275	9/11/2017	9/2/2024
SONG	2532	9/11/2017	14/10/2024
SPHR	1673	9/11/2017	9/6/2022
SPR	2532	9/11/2017	14/10/2024
START	2532	9/11/2017	14/10/2024
STV	1306	19/3/2021	14/10/2024
SUPER	1765	9/11/2017	12/9/2022
SXC	1199	4/7/2021	14/10/2024
SYS	2532	9/11/2017	14/10/2024
TAG	2520	9/11/2017	14/10/2024
THC	2532	9/11/2017	14/10/2024
TIPS	2532	9/11/2017	14/10/2024
TRC	3037	18/10/2015	9/2/2024
TROLL	2491	9/11/2017	14/10/2024
UBQ	2225	9/11/2017	12/12/2023
UFO	2188	9/11/2017	14/10/2024
UNIT	2532	9/11/2017	14/10/2024
UNO	2532	9/11/2017	14/10/2024
USNBT	1650	9/11/2017	16/5/2022
VIA	2532	9/11/2017	14/10/2024
VRC	1669	9/11/2017	4/6/2022
VTC	2532	9/11/2017	14/10/2024
WBB	2521	9/11/2017	14/10/2024
WDC	2759	18/10/2015	14/10/2024
XBC	2532	9/11/2017	14/10/2024
XCN	2532	9/11/2017	14/10/2024
XCO	2393	9/11/2017	28/5/2024
XCP	2532	9/11/2017	14/10/2024
XDN	2532	9/11/2017	14/10/2024
XEM	2532	9/11/2017	14/10/2024
XLM	2532	9/11/2017	14/10/2024
XMR	2532	9/11/2017	14/10/2024
XMY	2225	9/11/2017	12/12/2023
XPD	2532	9/11/2017	14/10/2024
XPM	2532	9/11/2017	14/10/2024
XPY	2517	9/11/2017	14/10/2024
XQN	2526	9/11/2017	14/10/2024
XRP	2532	9/11/2017	14/10/2024
XST	2349	9/11/2017	14/10/2024
XVG	2532	9/11/2017	14/10/2024
XWC	2532	9/11/2017	14/10/2024
ZET	2532	9/11/2017	14/10/2024
